# The prevalence of excessive weight in Balearic Islands’ young and middle-aged women and its association with social and socioeconomic factors: a ten-year trend (2000–2010)

**DOI:** 10.1186/s12889-015-2196-1

**Published:** 2015-09-02

**Authors:** Josep Ll. Coll, Maria del Mar Bibiloni, Rogelio Salas, Antoni Pons, Josep A. Tur

**Affiliations:** Research Group on Community Nutrition and Oxidative Stress, University of Balearic Islands, IdISPa, and CIBEROBN (Physiopathology of Obesity and Nutrition CB12/03/30038), Guillem Colom Bldg, Campus, E-07122 Palma de Mallorca, Spain; Faculty of Public Health Nutrition, Autonomous University of Nuevo León, 64460 Monterrey, Mexico

## Abstract

**Background:**

Knowledge about trends in the socioeconomic patterning of overweight and obesity in women provides insights into the nature of the obesity epidemic. Therefore the aim was to assess a ten-year trend (2000–2010) in the prevalence of excessive weight in Balearic Islands’ women and its association with socioeconomic factors.

**Method:**

Young (18–35 year-old) and middle-aged (36–55 year-old) women were selected from two population-based cross-sectional nutritional surveys carried out in the Balearic Islands, Spain. The participation rate was 80 % during 1999–2000 and 92.5 % during 2009–2010. Measured weight and height was obtained, and body mass index (kg/m^2^) was classified as follows: overweight (25.0 < 30), obese (≥30) and excessive weight (≥25). In both surveys, a general questionnaire including questions relating to socioeconomic status factors was used. Logistic regression was used to examine the association of excessive weight with socioeconomic variables and to test the interaction between the survey period and the socioeconomic factors.

**Results:**

Overall, while the prevalence of obesity mainly remained stable over the study period, the prevalence of overweight increased from 21.0 to 24.8 %. Young women showed an increased prevalence of overweight and excessive weight, from 14.1 to 20.9 % and from 20.9 to 28.6 %, respectively. Significant differences were not found in middle-aged women. Over the whole period, the incidence of excessive weight was higher among middle-aged and foreign women, but lower in women with a high educational profile and in employment. The prevalence of excessive weight in young women was also around 2.5 times higher in women who were living with at least one child at home. The tendency towards excessive weight in employed women decreased significantly between 2000 and 2010 in the younger age group (OR: 0.42; 95 % CI: 0.22–0.82).

**Conclusions:**

No significant increase in the prevalence of overweight/obesity was observed in middle-aged women, with a low level of education being the single socioeconomic variable associated with excessive weight in this target group. Overweight/obesity increased in young women with unemployment being the distinguishing socioeconomic factor associated with this increase.

## Background

Despite some signs suggesting that overweight and obesity are levelling off [1], it is a pandemic situation that poses one of the most serious public health challenges of the 21st century [2]. Decreasing quality of life [3, 4], rising serious chronic diseases (hypertension, diabetes, hypercholesterolemia, asthma, etc.) [5, 6] and predisposition to certain infections [7] are all associated with increased excessive weight (overweight + obesity). In addition, excessive weight also has financial consequences [8, 9]. For example, in 2008, the cost of obesity in the USA was estimated at around US$147 billion [10].

Socioeconomic factors have changed over time and, while obesity levels have been rising in all socioeconomic groups, some groups are more affected than others. Whereas, in the past, obesity was a disease of affluence, in recent decades it has been seen more frequently in lower socioeconomic groups [11], firstly in developed countries and afterwards in developing countries [12]. In this respect a consistent relationship between lower socioeconomic status and obesity has frequently been found in women [13]. Therefore, knowledge about trends in the socioeconomic patterning of overweight and obesity in women over time provides us with insights into the nature of the obesity epidemic.

In line with the remarkable socioeconomic changes observed in Europe over the first decade of the 21^st^ century, the Balearic Islands, an archipelago off the East Coast of Spain, has also been affected by cultural changes, the development of tourism, high population growth and an increase in immigration [14]. Persistent income-related problems with obesity have been reported in Spain [15], although the contribution of education has been considered to be the main socioeconomic variable explaining the prevalence of obesity [16]. It was pointed out recently that a country’s level of economic development changes the relationship between educational attainment and obesity, with stronger social patterning in women [17]. In Spain women showed a higher prevalence of obesity over time, but income-related inequalities were similar for both men and women in 1987. By 2006 figures remained relatively stable in men, whereas the inequality had tripled in women [18]. Therefore the aim of this study was to assess a ten-year trend (2000–2010) in the prevalence of excessive weight in Balearic Islands’ women and its association with socioeconomic factors (i.e. age, educational profile, professional profile, origin, cohabitation and living with at least one child at home).

## Methods

### Study design

The study was based on two population-based cross-sectional nutritional surveys carried out in the Balearic Islands, Spain (2000–2010).

### Study population, recruitment and approval

This study is part of the Balearic Islands’ Nutritional Survey (ENIB survey, 1999–2000) and the “Prevalence of Obesity in the Balearic Islands: their relationship with oxidative stress and inflammatory mediators” study (OBEX survey, 2009–2010). The ENIB and OBEX surveys were designed to obtain information on the health and nutritional status of the resident population in the Balearics [19–22]. In both surveys, the target population consisted of all inhabitants of the Balearic Islands aged between 16 and 65 years old. The population samples were taken from residents registered on the Balearic Islands’ official population census. The sampling techniques included stratification according to geographical area and municipality size, inhabitants’ age and gender and randomisation into subgroups, with the primary sampling units being Balearic municipal districts and individuals within these municipalities making up the final sample units.

### Sample selection

This analysis was limited to young (18–35 year-old) and middle-aged (36–55 year-old) women living in the Balearics with no missing data needed to calculate their body mass index (BMI, kg/m^2^). The participation rate was 80 % during 1999–2000 and 92.5 % during 2009–2010. Non-participation rates included potential subjects who declined to be interviewed (particularly over-55’s) as well as involuntary non-participants who were excluded due to unavoidable constraints on them taking part.

These sample sizes were considered sufficient to detect risk factors at regional level with 95 % confidence and a precision rate of 3.3 and 3.9 %, respectively. These sample sizes also calculated prevalence with 95 % confidence and a precision rate of 4.3 and 5.3 % in the young (*n* = 512) and middle-aged (*n* = 342) population in 1999–2000, respectively, and 5.0 and 6.2 % in the young (*n* = 378) and middle-aged (*n* = 252) in 2009–2010, respectively.

### Ethics

The study was conducted according to the guidelines laid down in the Declaration of Helsinki and all procedures were approved by the Balearic Islands Ethics Committee (approval reference numbers IB/135/98/PI and IB/1128/09/PI). Written informed consent was obtained from all subjects.

### Anthropometric measurements

Anthropometric measurements were performed by well-trained observers in order to avoid inter-observer coefficients of variation. Height was determined using a mobile anthropometer (Kawe 44444, Asperg, Germany) to the nearest millimetre, with the subject’s head in the Frankfurt plane. Body weight was determined to the nearest 100 g using a digital scale (Tefal, sc9210, Rumilly, France). Women were weighed in bare feet and light clothes, noting and subtracting the weight of the clothes. Weight and height measurements were used to calculate body mass index (BMI, kg/m^2^). Overweight, obesity and excessive weight were defined as BMI 25.0-29.9 kg/m^2^, ≥30 kg/m^2^, and ≥25.0 kg/m^2^, respectively [2].

### Socioeconomic determinants

In both surveys, a questionnaire including the following questions was used: age, region of origin (defined as being born in the Balearic Islands, the East of Spain as defined by the Mediterranean coast, other parts of Spain and other countries (in the OBEX survey the specific country was also included)), marital status (single, married, divorced, widowed and also “separated” in the OBEX survey), subjects living at home, educational profile (grouped according to years and type of education: low, <6 years at school; medium, 6 to 12 years’ education; high, 12 years’ education), and professional profile. The respondents were grouped into binary categories as follows: age group (young adults, 18–35 year-olds; middle-aged adults, 36–55 year-olds); origin (Spain; other countries); cohabitation (living alone, married or cohabiting); living with at least one child at home (none; yes); educational profile (low (including medium), i.e. ≤12 years of education; high, >12 years of education); and professional profile (unemployed, including student and homemaker; employed).

### Statistics

Analyses were performed with the SPSS statistical software package version 21.0 (SPSS Inc., Chicago, IL, USA). Significant differences in prevalence were calculated by means of χ^2^. Differences between group means were tested by an unpaired Students’ *t-*test. Logistic regression models with calculations of the corresponding odds ratio (OR) and a 95 % confidence interval (CI) were used to examine the possible association between socioeconomic factors (independent variables) and excessive weight (dependent variables). Binomial logistic regression analyses adjusted by age were first carried out for each socioeconomic variable and survey period. Binomial logistic regression analyses were also carried out for the prevalence of excessive weight including the socioeconomic variable, age (continuous variable), survey period and interaction between the two survey periods and the socioeconomic variable in order to examine the difference in prevalence between socioeconomic variables across time. The level of significance for acceptance was *P* < 0.05.

## Results

This study included 512 young (18–35 year-old) and 342 middle-aged (36–55 year-old) women living in the Balearics interviewed in the ENIB survey (1999–2000), and 378 young and 252 middle-aged women interviewed in the OBEX survey (2009–2010). Results revealed differences between the two surveys when it came to origin, educational profile, professional profile, cohabitation and living with at least one child (Table 1). Over the past decade an increase in the proportion of non-Spanish women living alone, with no children at home, with a high level of education and a job was observed. However, the rising proportion of women living alone and in employment was only shown in the middle-aged group. No significant changes were observed in the height of young women, but middle-aged women were noted to be taller. The prevalence of obesity mainly remained stable over the study period, but the prevalence of overweight increased from 21.0 to 24.8 %. Overweight and excessive weight also increased in young women, from 14.1 to 20.9 % and from 20.9 to 28.6 %, respectively. No statistically significant changes were found in the middle-aged group.

**Table 1:** Characteristics of the subjects^a,b^

	1999–2000 (ENIB survey)			2009–2010 (OBEX survey)		
	All	18–35	36–55	All	18–35	36–55
*n*	854	512	342	630	378	252
Weight (kg)^c^	62.1 ± 11.5	60.2 ± 11.0	65.0 ± 11.6	64.2 ± 11.9**	62.4 ± 12.4**	66.9 ± 13.3^NS^
Height (cm)^c^	161.3 ± 6.6	162.9 ± 6.1	159.0 ± 6.6	161.6 ± 6.4^NS^	162.1 ± 6.4^NS^	160.9 ± 6.5***
BMI (kg/m^2^)^c^	23.9 ± 4.5	25.7 ± 4.5	22.7 ± 4.1	24.6 ± 4.9**	25.9 ± 5.2^NS^	23.7 ± 4.4***
Prevalence of overweight (%)^d^	21.0	14.1	31.3	24.8*	20.9**	30.6^NS^
Prevalence of obesity (%)^d^	11.1	6.8	17.5	11.0^NS^	7.7^NS^	15.9^NS^
Prevalence of excessive weight^1^ (%)^d^	32.1	20.9	48.8	35.7^NS^	28.6**	46.4^NS^
Origin (%)^d^						
Spanish	95.0	96.5	92.7	84.9***	82.7***	88.3*
Non-Spanish	5.0	3.5	7.3	15.1	17.3	11.7
Cohabitation (%)^d^						
Living alone	37.0	58.2	5.3	42.7*	62.9^NS^	12.4**
Married or cohabiting	63.0	41.8	94.7	57.3	37.1	87.6
Living with at least one child at home (%)^d^						
None	51.2	70.9	21.6	63.8***	84.6***	32.7**
Yes	48.8	29.1	78.4	36.2	15.4	67.3
Educational profile (%)^d^						
Low (including medium)	76.5	67.9	89.8	58.0***	58.9**	56.6***
High	23.5	32.1	10.2	42.0	41.1	43.4
Professional profile (%)^d^						
Unemployed	40.5	39.1	42.8	28.8***	38.1^NS^	14.9***
Employed	59.5	60.9	57.3	71.2	61.9	85.1

*BMI* body mass index, *NS* not significant

^a^Prevalence of overweight: BMI ≥25- < 30 kg/m^2^; obesity: ≥30 kg/m^2^; excessive weight: BMI ≥ 25 kg/m^2^

^b^Data were expressed as ^c^mean ± standard deviation and ^d^prevalence (%). Differences between surveys were tested by ^c^unpaired Students’ t-test and ^d^χ^2^: *P < 0.05, **P < 0.01, ***P < 0.001

In both surveys, adjusted analysis with socioeconomic variables (Table 2) showed an increased risk of excessive weight in middle-aged and non-Spanish women and a decreased risk in women with a high educational profile. In 2009–2010, the prevalence of excessive weight was also 0.5 times lower amongst women in employment. In young women (Table 3), the prevalence was around 2.5 times higher in women who were living with at least one child at home. Educational profile was the only socioeconomic variable associated with the risk of excessive weight or obesity in middle-aged women (Table 4). Overall, the probability of being overweight decreased significantly between 2000 and 2010 in both middle-aged and employed women. However, the association with employment only decreased significantly in the younger group.

**Table 2:** Global results of binomial regressions of excessive weight prevalence by social and socioeconomic factors and survey period in Balearic Islands’ women^a,b,c^

	1999–2000 (ENIB survey)		2000–2010 (OBEX survey)			
	BMI ≥ 25 *n* (%)	Adjusted OR (95 % CI)^b^	BMI ≥ 25 *n* (%)	Adjusted OR (95 % CI)^b^	Change in Prevalence (%)^c^	*P*-value for interaction^c^
*n*	274 (32.1)		225 (35.7)			
Age group						
18–35 years	107 (39.1)	1.00 (ref.)	108 (48.0)	1.00 (ref.)	1.00 (ref.)	
36–55 years	167 (60.9)	3.61 (2.67, 4.88)***	117 (52.0)	2.16 (1.55, 3.02)***	0.60 (0.38, 0.94)	0.026
Educational profile						
Low (including medium)	246 (89.7)	1.00 (ref.)	149 (66.1)	1.00 (ref.)	1.00 (ref.)	
High	28 (10.3)	0.42 (0.27, 0.66)***	76 (33.9)	0.52 (0.37, 0.75)***	1.26 (0.71, 2.24)	0.436
Professional profile						
Unemployed	117 (42.7)	1.00 (ref.)	69 (30.6)	1.00 (ref.)	1.00 (ref.)	
Employed	157 (57.3)	0.88 (0.64, 1.20)	156 (69.4)	0.50 (0.33, 0.76)**	0.56 (0.34, 0.92)	0.022
Origin						
Spanish	255 (93.1)	1.00 (ref.)	181 (80.4)	1.00 (ref.)	1.00 (ref.)	
Non-Spanish	19 (6.9)	1.07 (1.05, 1.08)***	44 (19.6)	1.06 (1.04, 1.07)***	1.53 (0.69, 3.40)	0.297
Cohabitation						
Living alone	60 (21.9)	1.00 (ref.)	73 (32.4)	1.00 (ref.)	1.00 (ref.)	
Married or cohabiting	214 (78.1)	1.03 (0.67, 1.59)	152 (67.6)	1.05 (0.69, 1.59)	0.83 (0.51, 1.36)	0.465
Living with at least one child at home						
None	101 (36.9)	1.00 (ref.)	121 (53.8)	1.00 (ref.)	1.00 (ref.)	
Yes	173 (63.1)	1.15 (0.81, 1.63)	104 (46.2)	1.15 (0.77, 1.70)	0.86 (0.54, 1.36)	0.519

^a^Prevalence of excessive weight: BMI ≥ 25 kg/m^2^

^b^Binomial logistic regression analysis adjusted by age. *P < 0.05, **P < 0.01, ***P < 0.001

^c^Binomial logistic regression analysis adjusted by age (continuous) including the social and socioeconomic factors, survey periods and the interaction between the survey periods and the socioeconomic factor. *P < 0.05, **P < 0.01, ***P < 0.001

**Table 3:** Results of binomial regressions of excessive weight prevalence by social and socioeconomic factors and survey period in young Balearic Islands’ women (18–35 years-old)^a,b,c^

	1999–2000 (ENIB survey)		2000–2010 (OBEX survey)			
	BMI ≥ 25 *n* (%)	Adjusted OR (95 % CI)	BMI ≥ 25 *n* (%)	Adjusted OR (95 % CI)	Change in Prevalence (%)	*P*-value for interaction
*n*	107 (20.9)		108 (28.6)			
Educational profile						
Low (including medium)	88 (81.9)	1.00 (ref.)	70 (64.8)	1.00 (ref.)	1.00 (ref.)	
High	19 (18.1)	0.43 (0.25, 0.74)**	38 (35.2)	0.47 (0.28, 0.78)**	1.17 (0.56, 2.46)	0.682
Professional profile						
Unemployed	32 (29.9)	1.00 (ref.)	48 (44.3)	1.00 (ref.)	1.00 (ref.)	
Employed	75 (70.1)	1.25 (0.74, 2.09)	60 (55.7)	0.45 (0.27, 0.77)**	0.42 (0.22, 0.82)	0.010
Origin						
Spanish	100 (93.5)	1.00 (ref.)	81 (75.0)	1.00 (ref.)	1.00 (ref.)	
Non-Spanish	7 (6.5)	2.06 (0.77, 5.51)	27 (25.0)	2.15 (1.21, 3.82)**	1.05 (0.34, 3.27)	0.938
Cohabitation						
Living alone	50 (46.7)	1.00 (ref.)	61 (56.5)	1.00 (ref.)	1.00 (ref.)	
Married or cohabiting	57 (53.3)	1.26 (0.70, 2.27)	47 (43.5)	1.13 (0.67, 1.88)	0.91 (0.48, 1.72)	0.774
Living with at least one child at home						
None	58 (54.2)	1.00 (ref.)	241 (73.1)	1.00 (ref.)	1.00 (ref.)	
Yes	49 (45.8)	2.40 (1.28, 4.52)**	29 (26.9)	2.56 (1.39, 4.71)**	1.27 (0.61, 2.64)	0.526

^a^Prevalence of excessive weight: BMI ≥ 25 kg/m^2^

^b^Binomial logistic regression analysis adjusted by age. *P < 0.05, **P < 0.01, ***P < 0.001

^c^Binomial logistic regression analysis adjusted by age (continuous) including the social and socioeconomic factors, survey periods and the interaction between the survey periods and the socioeconomic factor. *P < 0.05, **P < 0.01, ***P < 0.001

**Table 4:** Results of binomial regressions of excessive weight prevalence by social and socioeconomic factors and survey period in middle-aged Balearic Islands’ women (36–55 years-old)^a,b,c^

	1999–2000 (ENIB survey)		2000–2010 (OBEX survey)			
	BMI ≥ 25 *n* (%)	Adjusted OR (95 % CI)	BMI ≥ 25 *n* (%)	Adjusted OR (95 % CI)	Change in Prevalence (%)	*P*-value for interaction
*n*	167 (48.8)		117 (46.4)			
Educational profile						
Low (including medium)	158 (94.9)	1.00 (ref.)	79 (67.2)	1.00 (ref.)	1.00 (ref.)	
High	9 (5.1)	0.39 (0.17, 0.90)*	38 (32.8)	0.55 (0.32, 0.95)*	1.37 (0.51, 3.69)	0.538
Professional profile						
Unemployed	61 (50.9)	1.00 (ref.)	21 (18.1)	1.00 (ref.)	1.00 (ref.)	
Employed	114 (49.1)	0.65 (0.41, 1.03)	96 (81.9)	0.58 (0.28, 1.20)	0.85 (0.36, 2.01)	0.718
Origin						
Spanish	162 (92.8)	1.00 (ref.)	100 (85.2)	1.00 (ref.)	1.00 (ref.)	
Non-Spanish	13 (7.2)	1.09 (0.48, 2.50)	117 (14.8)	1.97 (0.87, 4.48)	1.71 (0.54, 5.45)	0.366
Cohabitation						
Living alone	8 (6.0)	1.00 (ref.)	12 (10.3)	1.00 (ref.)	1.00 (ref.)	
Married or cohabiting	167 (94.0)	0.68 (0.25, 1.81)	105 (89.7)	1.11 (0.49, 2.48)	1.76 (0.50, 6.22)	0.382
Living with at least one child at home						
None	31 (25.7)	1.00 (ref.)	42 (35.9)	1.00 (ref.)	1.00 (ref.)	
Yes	144 (74.3)	0.74 (0.43, 1.26)	75 (64.1)	0.78 (0.45, 1.35)	1.02 (0.47, 2.019)	0.964

^a^Prevalence of excessive weight: BMI ≥ 25 kg/m^2^

^b^Binomial logistic regression analysis adjusted by age. *P < 0.05, **P < 0.01, ***P < 0.001

^c^Binomial logistic regression analysis adjusted by age (continuous) including the social socioeconomic factors, survey periods and the interaction between the survey periods and the socioeconomic factor. *P < 0.05, **P < 0.01, ***P < 0.001

## Discussion

The general findings of this study are as follows. Firstly, over the past decade the female population in the Balearic Islands has undergone social and socioeconomic changes, with an increase in the proportion of non-Spanish women, an increase in women living alone –particularly in the middle-aged group–, with no children at home, with a high educational profile and also in employment, with the latter being particularly noticeable in the middle-aged group. Secondly, in line with these social and socioeconomic changes the overall prevalence of overweight increased. An increased prevalence in overweight and excessive weight was observed in young women, although no changes were seen in the middle-aged group. Thirdly, in both surveys the overall risk of excessive weight was associated with age, origin, educational profile and also the likelihood of having a job in 2009–2010. Living with at least one child at home was also associated with the risk of excessive weight in young women and a low educational profile was the only identified risk factor in middle-aged women. Finally, the probability of middle-aged employed women having excessive weight decreased significantly over time.

Worldwide, the age-standardised prevalence of excessive weight increased from 24.6 % (95 % uncertainty interval (UI): 22.7–26.7 %) in 1980 to 34.4 % (95 % UI: 33.2–35.5 %) in 2008 [23]. Moreover, age-standardised obesity prevalence nearly doubled from 6.4 % (95 % UI: 5.7–7.2) to 12.0 % (95 % UI: 11.5–12.5) during the same 28 year period [23]. Half of this change occurred during the 1980–2000 period and the other half occurred during 2000–2008 [23]. The regions with reported increases in the prevalence of obesity were Central Latin America, South Latin America and Oceania [23]. In women, the worldwide proportion with excessive weight increased from 29.8 % (95 % UI: 29.3–30.2) in 1980 to 38.0 % (UI: 37.5–38.5 %) in 2013 [24]. In 1990, more than 50 % of women had excessive weight in 47 countries, a figure that increased to 74 in 2000 and to 101 in 2008 [23]. In Europe, over the 2000–2009 period, obesity and excessive weight in adult women ranged between 13.3 and 30.0 and 31.0 and 53.2 %, respectively [25]. Our results back up the results of a previous Spanish study that revealed an increased prevalence in excessive weight of 10.3 % among women over the 1987–2007 period [26]. Nevertheless, contrary to Spanish literature [27] which positioned Spain as one of the countries where the occurrence of obesity has risen most substantially, our results did not reveal an increased prevalence of obesity in young and middle-aged women.

Increased BMI is associated with age [28, 29], with the greatest weight gain occurring between the ages of 20 and 40 [24]. Young women showed the largest rising occurrence of excessive weight, which agrees with previous studies [30, 31]. Howel [32] described trends (1999–2006) in the prevalence of obesity and overweight in English adults and also found that when the subjects were divided into 10-year pseudo-birth-cohorts, the prevalence of obesity and overweight was consistently higher at a given average age for pseudo-cohorts born more recently.

Furthermore, to our knowledge, few studies have determined trends in excessive weight by evaluating its association with socioeconomic factors [33, 34]. Moreover, controversial results have been found in literature due to differences in the anthropometric measurements used (i.e. self-reported *vs.* measured data), socioeconomic indicators, time periods and/or statistical analysis [11].

### Educational profile

In Western societies an inverse relationship between educational profile and excessive weight has been shown many times, especially amongst women [13]. A comparative appraisal of educational inequalities in overweight and obese adults in 19 European countries found the largest educational inequalities in Mediterranean women [35]. Overweight and/or obesity increases have also been associated with low levels of education in Swiss men and women [34], English women [11] and USA men [36]. An increase in the proportion of obesity in Spanish women associated with a low level of education was also reported between 1987 and 1997 [37]. Spanish people with a lower educational profile showed lower energy intake [38], just as women with a lower educational profile showed sedentary behaviour during their leisure time [39].

### Employment status

Previous studies have also associated unemployment with poor health [40–42] such as obesity [43], mortality from cardiovascular diseases [44] and infectious diseases [45]. However, few studies have investigated trends in excessive weight according to occupational profile [46, 47]. Knowledge about trends in excessive weight for women from different occupational profiles provides us with insights into the nature of obesity [48]. The occupational transition undergone by women, from unemployed status to the various current professional profiles (agriculture-production, clerical, services and management), is associated with a lower excessive weight prevalence [46]. However, the international financial crisis, which emerged in full force after September 2008, had a hugely negative impact on Spain where there was a large increase in unemployment. It is estimated that 1.02 million young adults aged between 20 and 34 were inactive in the last quarter of 2010 [49]. At present, in the first quarter of 2015, 0.96 million young adults were still inactive and this figure may have decreased to 0.73 million [49]. Nevertheless, in recent years, time spent in education has increased in Spain. In 2000 some 23 % of 25–64 year-olds and 34 % of 25–34 year-olds were enrolled in tertiary education and by 2011, 32 % of 25–64 year-olds and 39 % of 25–34 year-olds were enrolled in tertiary education [50]. The percentage of young people (15–29 year-olds) not in employment but in tertiary level education also increased from 12.7 in 2008 to 21.4 % in 2011, which suggests that some young Spaniards see education as a temporary way out of unemployment and a potential advantage when they try to get back into employment at a later stage [50]. Our results suggest that there may have been an increase in BMI differences between employed and unemployed young adults; however, further research focussing on this possible relationship is needed.

### Origin

Traditionally Spain was an emigrant country and only became a host country during the last century. Specifically, in the Balearic Islands, the number of people from abroad has increased by 228.7 % (i.e. 3.2-fold) [51]. The trend throughout the western world as well as in most other parts of the world is that height is increasing in both women and men [52]. However, no significant changes were observed in young women, but height was seen to have increased in middle-aged women. These results may be attributed to the higher proportion of women born in countries other than Spain, mainly young women (5 % in 1999–2000 and 15.1 % in 2009–2010; data not shown). On the other hand, although some previous studies have associated migration with obesity [53, 54] and cardiovascular risks [55, 56], no trend in excess weight linked to origin was found in this study. Low social support, stress, the price and size of meals and shopping, cooking and eating habits are all linked to higher excessive weight in the foreign population [57]. However, literature also links migration to a protective effect against the risk of obesity [58–60]. For example, in the USA, adult immigrants showed a lower tendency towards being overweight and obese than their USA-born counterparts [61], but there is a tendency for this protective effect to dissipate depending on the length of stay [60, 62] when the risk of excessive weight increases [54, 60, 63, 64].

### Family and marital status

Although no trend towards excesive weight linked to family and marital status was found in this study, it has previously been reported in literature that unmarried subjects are less likely to have excessive weight than married subjects [65–69]. The mechanism that links marital status and excessive weight is not so clear. It may well be due to the social obligations of marriage or a lack of concern about being attractive, which is particularly common among women. Nevertheless, literature has also previously reported that eating as a family protects young people and adults against obesity [70].

### Strengths and limitations

A major methodological strength of this study is the anthropometric method used to obtain weight and height, which were measured by trained personnel. The interaction between the survey period and socioeconomic factors on excessive weight prevalence were assessed by binary logistic regression analyses applying the hierarchy principle (i.e. including the survey period, the socioeconomic factor and also the survey socioeconomic factor in the models) and only adjusted by age (continuous variable). Nevertheless, the use of large data sets is a major strength of this study.

The study also has several limitations which should be taken into consideration when interpreting its findings. Firstly, the cross-sectional designs provided no basis for studying causality. Longitudinal data would provide a more valid and reliable estimate of the prevalence of excessive weight. Secondly, the unemployed category was heterogeneous and more detailed information would have been beneficial to the study. Thirdly, there are few explanatory variables in this study with, for example, no exploration of physical activity levels or dietary behaviour.

## Conclusion

To sum up, no significant increase in the prevalence of overweight/obesity was observed among middle-aged women and a low level of education was the single socioeconomic variable associated with excessive weight in this target group. In contrast, a significant increase in the prevalence of excessive weight was observed in young women and the single socioeconomic variable associated with this increase was unemployment. Factors relating to differences in the rate of increase of excessive weight in adult women will need further research.

## Abbreviations

ENIB survey: Balearic Islands’ Nutritional Survey
OBEX survey: “Prevalence of Obesity in the Balearic Islands: their relationship with oxidative stress and inflammatory mediators” study
BMI: Body mass index
OR: Odds ratio
CI: Confidence interval
UI: Uncertainty interval
